# Whole genome sequencing study of identical twins discordant for psychosis

**DOI:** 10.1038/s41398-024-02982-0

**Published:** 2024-07-30

**Authors:** Cathal Ormond, Niamh M. Ryan, Anna M. Hedman, Tyrone D. Cannon, Patrick F. Sullivan, Michael Gill, Christina Hultman, Elizabeth A. Heron, Viktoria Johansson, Aiden Corvin

**Affiliations:** 1 Neuropsychiatric Genetics Research Group, Department of Psychiatry, Trinity College Dublin, Dublin, Ireland; 2 Department of Epidemiology and Biostatistics, Karolinska Institutet, Stockholm, Sweden; 3 Departments of Psychology and Psychiatry, Yale University School of Medicine, New Haven, CT USA; 4 Departments of Genetics and Psychiatry, University of North Carolina, Chapel Hill, NC USA; 5 Department of Clinical Sciences, Psychiatry Unit, Umeå University, Umeå, Sweden

**Keywords:** Genomics, Schizophrenia, Bipolar disorder

## Abstract

Monozygotic (MZ) twins are often thought to have identical genomes, but recent work has shown that early post-zygotic events can result in a spectrum of DNA variants that are different between MZ twins. Such variants may explain phenotypic discordance and contribute to disease etiology. Here we performed whole genome sequencing in 17 pairs of MZ twins discordant for a psychotic disorder (schizophrenia, schizoaffective disorder or bipolar disorder). We examined various classes of rare variants that are discordant within a twin pair. We identified four genes harboring rare, predicted deleterious missense variants that were private to an affected individual in the cohort. Variants in *FOXN1* and *FLOT2* would have been categorized as damaging from recent schizophrenia and bipolar exome sequencing studies. Additionally, we identified four rare genic copy number variants (CNVs) private to an affected sample, two of which overlapped genes that have shown evidence for association with schizophrenia or bipolar disorder. One such CNV was a 3q29 duplication previously implicated in autism and developmental delay. We have performed the largest MZ twin study for discordant psychotic phenotypes to date. These findings warrant further investigation using other analytical approaches.

## Introduction

Schizophrenia is a substantially heritable brain disorder, with heritability estimated between 60–80% [1]. The core symptoms include psychosis, where perception of reality is impaired, and people develop delusions or hallucinations [2]. The genetic etiology is complex, and known to overlap significantly with other psychiatric disorders, particularly those that present with psychosis, e.g., schizoaffective disorder, bipolar disorder [3, 4]. Despite significant progress, much of the genetic variation contributing to risk of schizophrenia and related psychotic disorders remains to be determined [5]. The search has moved from array-based studies, which focus on common variants in genome-wide association studies (GWAS), to whole exome (WES) or genome (WGS) sequencing approaches which provide more complete analysis of the full spectrum of genetic variation [6, 7]. This is important, as rare mutations that disrupt gene function, identifiable by sequencing, may be particularly informative in understanding the molecular etiology involved, and provide targets for novel therapies. Because deleterious alleles with large effects tend to be removed over time by natural selection, they tend to be rare in the population and hence very large sample sizes are required to identify them using case-control studies [8, 9].

While the rare variant case-control approach has yielded success in identifying robust risk genes for psychotic disorders [10], there are other potential analytical strategies available. Historically, twin research has played an important role in revealing the genetic epidemiology of psychiatric disorders. Heritability estimates from twin studies for schizophrenia [11] and bipolar disorder [12] are substantially higher than the heritability explained from GWAS [13, 14], so it is likely that other genetic factors (e.g. rare variants) are contributing to the heritability. Concordance rates between monozygotic (MZ) twins for schizophrenia is estimated to be 50% [15], which is notably higher than the approximately 1% incidence rate in the general population [16]. The case co-twin study design allows for the control of shared genetic and environmental effects and for the examination of non-shared genetic variation. Postnatal environmental effects such as childhood trauma or substance abuse are known to increase risk for a psychotic disorder [17]. While MZ twins are the same age and often have similar childhood experiences, it is possible that they may not share such environmental effects.

One hypothesis explaining the discordance in diagnosis between MZ twins is that both individuals share a common genetic and environmental risk which is insufficient alone to be causal for the phenotype, but rare, post-zygotic genetic variation present in the affected twin increases their disease-risk. A recent study estimated that in general, almost 10% of de novo variants occur post-fertilization and prior to progenitor germ cell specification and were thus likely to be present in both germ and blood cells [18]. Another study examined transmission of post-zygotic variants to offspring of monozygotic twins and estimated that 2.1% of de novo variants occurred after the twinning event, but prior to progenitor germ cell specification [19]. These discordant de novo variants would form part of what is estimated from twin or family studies as the environmental or even non-additive genetic effects. Given their rarity, examining variation private to one twin will drastically reduce the search space of candidate causal variants compared to unrelated case-control cohorts. WGS and WES analyses have identified de novo post-zygotic variation in MZ twins discordant for a range of disorders [20–22] and further investigation in psychotic disorders is warranted. Such knowledge is useful from a clinical perspective, as it highlights another important factor that may be responsible for the discordance in clinical diagnoses between MZ twins.

Here we performed WGS on peripheral blood samples from 17 pairs of MZ twins discordant for schizophrenia, schizoaffective disorder, or bipolar disorder, which is the largest sample size for such a study to date. Using a strict filtering and annotation approach, we have identified discordant single nucleotide variants (SNVs) and copy number variants (CNVs) that may provide novel insights into the genetic basis of these conditions.

## Methods and materials

### Sample procurement

#### Ethics

Written informed consent was obtained from all participants in this study. Ethical permissions were obtained from Stockholm County in Sweden (Dnr: 2004/448/4, 2007/779-31/3 and 2008/292-32).

#### Recruitment and diagnostic assessment procedures

The schizophrenia and bipolar twin study in Sweden (STAR) is a study on MZ and DZ twin pairs with schizophrenia or bipolar disorder and in total 462 twins have participated, see Johansson et al. for further description of the cohort [23]. The participants in this study were originally identified through the Swedish Twin register (STR) [24] and the National Patient register (NPR), which is administered by the Social board of health and welfare. Potential participants were invited to the STAR study if only one twin had a registered treatment episode of schizophrenia or bipolar disorder (Diagnoses according to International Statistical Classification of Diseases: ICD-8: 295 or 296, ICD-9: 295 or 296 or ICD-10: F20, F30 or F31). If the twin pair decided to participate in STAR, an extensive assessment procedure was initiated. Diagnostic status was confirmed by a clinical psychiatrist through the Structured Clinical Interview for DSM-IV (SCID-I) [25]. The final diagnosis was determined by an evaluation team, with access to register data from previous hospitalizations and hospital records. The diagnoses were categorized as schizophrenia (ICD-10: F20), schizoaffective disorder (ICD-10: F25), bipolar disorder (ICD-10: F31), major depressive disorder (ICD-10 F32-F33) or not affected by any of those diagnoses.

From the STAR cohort we selected all available disease discordant MZ twin pairs (*n* = 19). We included pairs in which one twin was affected from schizophrenia, schizoaffective disorder, or bipolar disorder, and the co-twin was not affected from any of those diagnoses. These diagnoses were selected based on the shared genomics from rare variants [26] and given that all three can exhibit psychotic symptoms. In addition, schizoaffective disorder is often included in the case definition for both schizophrenia and bipolar disorder analyses [10, 26]. Rare, protein altering variants have a more modest effect on major depressive disorder (MDD) without psychosis or bipolar disorder [27] compared to schizophrenia [10] or bipolar disorder [26]. In addition, the shared common variant heritability between MDD and schizophrenia (r_g_ = 0.37) or between MDD and bipolar disorder (r_g_ = 0.34) are weaker than the shared heritability between schizophrenia and bipolar disorder (r_g_ = 0.68) [28]. We therefore included pairs where the co-twin had a diagnosis of MDD without occurrence of psychotic symptoms and considered that individual as unaffected.

### Whole genome sequencing and quality control

#### DNA collection and extraction

The same day as the clinical assessment, blood samples for DNA extraction were collected in the morning and were sent to Karolinska Institutet (KI) Biobank for processing and storage. DNA was extracted from EDTA blood based on a salting out method from Puregene extraction kit using a Gentra robot. DNA concentrations were quantified by Qubit and the quality of DNA was determined by agarose gel electrophoresis. Both samples from the pair T19 failed quality control metrics for sequencing and were excluded.

#### Sequencing and quality control

WGS was performed by Edinburgh Genomics (Clinical Genomics) on a HiSeqX to an average depth of coverage of 30x per sample. WGS allowed us to examine non-coding regions which would be largely inaccessible from whole exome sequencing data. Additionally, WGS can give us better breakpoint resolution for calling CNVs. All FASTQ files were examined using *FastQC* and *samtools* [29] to identify DNA contamination or degradation. Reads were aligned to the GRCh38 reference genome using *BWA-MEM* [30], following the GATK Best Practices [31]. Briefly, this involved marking PCR duplicates, base quality score recalibration, local realignment of reads around indels, and variant calling with HaplotypeCaller (GATK version 3.8-0-ge9d806836). Genotype calling was performed jointly across all samples, and variant quality score recalibration (VQSR) was performed on the SNVs and Indels separately (see Supplementary Methods).

The software *peddy* [32] was applied to all samples jointly to check for: (i) relatedness discordance; (ii) sex discordance; (iii) low median coverage; and (iv) ancestry clustering by a principal component analysis (PCA) based on 1000 Genomes Project data [33]. Sample T04_U was flagged as having a relatedness error with all other samples at this stage, and so twin pair T04 was excluded. SNP array data was available for 35 samples, and the genotype concordance rates between the sequence data and SNP array data was determined using the *GenotypeConcordance* module from GATK. To confirm zygosity within each twin pair, the genotype concordance rates for the sequence data and for the SNP array data were determined using *picard* and GATK respectively (see Supplementary Methods).

### SNV and indel prioritization

To remove low-quality variants, any variant with QUAL < 100.0 was removed across all samples [22]. In addition, if any sample had GQ < 20.0 or DP < 10.0, the genotype for that sample was set to missing [34]. The variants of interest are putative de novo events present in a twin pair, (i.e., where the affected sample had exactly one more copy of the allele of interest than their co-twin). A reverse-pairwise analysis was performed within each twin pair to identify such variants, regardless of affectation status. The Variant Effects Predictor (VEP) [35] was used to annotate each entry for: functional impact (sequence ontology) [36]; predicted deleteriousness (SIFT [37], PolyPhen-2 [38] and CADD [39]), and allele frequency (1000 Genomes Project [40] and gnomAD [41]).

To identify rare, putatively pathogenic variants, the following filters were applied: (i) variants were present in the coding sequence of a protein coding gene as determined by RefSeq [42]; (ii) VEP impact was MODERATE or HIGH; (iii) SIFT was “deleterious” or PolyPhen was “damaging”; (iv) the allele frequency was <1% or absent in the appropriate population groups in the 1000 Genomes Project and gnomAD databases; and (v) variants were not observed in any other twin pair within the cohort. Only SNVs were considered at this step, as SIFT and PolyPhen do not provide scores for indels. Multi-allelic sites were split to bi-allelic sites to further identify the pathogenic allele.

### CNV calling and prioritization

Germline CNVs were called using a family-based consensus using four separate calling tools (Supplementary Methods). All the calls within a twin pair were combined (Supplementary Figs. 1, 2) to create Regions of Interest (ROIs). Any ROI that was found in one sample of the pair and identified by only one calling algorithm was removed. Once a list of high-confidence ROIs has been generated, variants were removed if they had at least a 50% reciprocal overlap with any common CNVs (i.e. frequency at least 1% in the appropriate population group) in the following public databases: gnomAD [41], the Deciphering Developmental Disorders (DDD) study [43], and the Database of Genomic Variants (DGV) [44]. As the DDD and gnomAD databases were curated relative to the hg19 genome build, the ROI files were converted to this build using the UCSC liftOver tool [45].

Any ROI that had a 50% reciprocal overlap with a variant labelled as “Pathogenic” in the NIH Clinical Genomics (ClinGen) CNV database (UCSC “iscaPathogenic” table) [46] was retained, regardless of population frequency. Since ClinGen collates CNV calls from a wide collection of sources, each of which may use different reference material for CNV calling, it is not possible to know if the type of pathogenic CNV matches that of the CNV call in our data. Hence, CNV calls were not matched for type at this stage. Pathogenic CNVs were retained if the associated phenotype was psychiatric or neurodevelopmental in nature.

## Results

### WGS data

WGS was performed on 19 MZ twin pairs discordant for a psychotic disorder, to a minimum of 30x coverage (Methods). After quality control, 17 pairs of twins were carried forward for analysis (Table 1, Supplementary Table 1). Principal components analysis identified that all samples were of European ancestry with the exception of twin pair T05 who were from East Asia (Supplementary Figs. 3–5). The within-pair genotype concordance rates confirmed the expected zygosity (Supplementary Methods; Supplementary Table 2). A high concordance between the WGS data and previously generated genotype array data for each sample confirmed that there was no sample mix-up. After removing lower quality variants, each sample had an average of 44 306 (standard deviation: 1640.5; median: 44,264; range: 41,134–48,050) discordant SNVs and indels across the genome (Supplementary Table 1). Given that short read WGS detects approximately 4,000,000 variants per genome [47], this implies approximately 1% of the variants detected in each sample are discordant. As the estimated error rate is 0.1% for short-read WGS on Illumina HiSeq technologies [48], this number is unlikely to be attributed solely to sequencing errors.

**Table 1:** Phenotypic data for the 17 pairs of MZ twins.

Twin Pair ID	Sex	Age at Sampling	Genomic Ancestry	Twin 1 Phenotype	Twin 2 Phenotype	Discordance
T01	M	35	European	SCZ	MDD	Broad
T02	M	38	European	SAD	None	Narrow
T03	F	34	European	SCZ	None	Narrow
T05	M	25	East Asian	SAD	MDD	Broad
T06	F	65	European	SAD	None	Narrow
T07	F	61	European	BD	None	Narrow
T08	F	60	European	SAD	None	Narrow
T09	F	59	European	BD	None	Narrow
T10	M	58	European	SCZ	None	Narrow
T11	F	52	European	BD	None	Narrow
T12	M	50, 51	European	BD	None	Narrow
T13	M	48	European	SCZ	MDD	Broad
T14	M	50, 51	European	BD	None	Narrow
T15	M	43	European	SAD	MDD	Broad
T16	M	46	European	BD	None	Narrow
T17	F	45	European	BD	None	Narrow
T18	F	27	European	SAD	MDD	Broad

For the discordance, “broad” indicates that both samples have a diagnosis and “narrow” indicates that only one sample has a diagnosis.

*SCZ* schizophrenia, *MDD* major depressive disorder, *SAD* schizoaffective disorder, *BD* bipolar disorder.

### Discordant SNVs and indels

To identify rare, putatively pathogenic variants that may have a direct effect on disease burden, a rigorous filtering pipeline was applied to discordant SNVs present in protein-coding regions (Methods). After applying filters, six rare, predicted deleterious discordant SNVs were discovered across four unique genes, (Table 2). For *FOXN1*, three discordant variants present in close proximity ( < 15 bp) in the same individual appeared on the same re-constructed reads, likely due to re-alignment of reads around indels during variant calling (Supplementary Fig. 6). We used CADD scores [39] and the RegulomeDB database [49] to examine rare, discordant, putatively pathogenic variants with a predicted regulatory effect (Supplementary Methods). All variants were required to have a Phred-scaled CADD score > 20.0 (i.e., in the top 1% of predicted deleterious variants across the genome) and a RegulomeDB score of 1 or 2. One such variant was identified but was present in an unaffected individual (Supplementary Table 4).

**Table 2:** Discordant protein-coding variants with a predicted pathogenic effect.

Chr	Pos	rsID	Ref	Alt	Sample	Phenotype	Gene	HGVSp	SIFT	PolyPhen
chr9	96932219	rs112610837	C	T	T13_A1	SCZ	*NUTM2G*	P172S	T	D
chr17	28530789	rs1385768054	A	C	T07_A	BD	*FOXN1*	S291R	D	D
chr17	28530791	rs371766542	C	A	T07_A	BD	*FOXN1*	S291R	D	D
chr17	28530802	rs1220808552	G	C	T07_A	BD	*FOXN1*	S295T	D	D
chr17	28881251	-	C	T	T09_A	BD	*FLOT2*	A347T	D	D
chr22	44592351	rs367621282	G	C	T10_A	SCZ	*KRTAP10-6*	P45R	D	D

Each variant is annotated with genomic positions (GRCh38); rs identification numbers; the reference and alternative alleles of the variant; the sample carrying the variant; the phenotype of the sample (*SCZ* schizophrenia, *BD* bipolar disorder); the gene harboring the variant; the amino acid substitution (HGVSp); and pathogenicity scores from SIFT (*D* deleterious, *T* tolerated) and PolyPhen (*D* damaging). All prioritized variants are missense SNVs.

We considered eight regulatory annotation features likely to have an impact on phenotype, derived from the ENCODE project as well as brain specific annotation features (Supplementary Methods). We use a Wilcoxon signed-rank sum test to evaluate whether the median count of all discordant variants overlapping each feature was different between the affected samples and the unaffected samples within a twin pair (Supplementary Fig. 7). No significant difference in median counts were observed for any regulatory feature (Supplementary Table 5).

### CNVs and repeat expansions

Germline CNVs were called using a consensus approach based on four separate calling algorithms, extended to take family structure into account (Methods, Supplementary Methods). We screened all individuals against a list of 23 rare CNVs (Supplementary Table 6) previously implicated in schizophrenia or related psychotic disorders [50, 51]. One of these, a duplication on chromosome 13q12.11, was identified in both samples of twin pair T09. This CNV had previously been reported as having a protective effect for schizophrenia.

We prioritized discordant CNVs in cases, that were either rare/absent in public databases or had a known pathogenic effect. After applying filters, four rare CNVs that overlapped gene regions were identified across the cohort (Table 3). Of note, a duplication on chromosome 3q29 was observed in the affected sample of twin pair T17. While 3q29 deletions have been associated with schizophrenia, 3q29 duplications have been implicated in autism spectrum disorders and developmental delay [52]. This prompted us to examine a more extensive list of CNVs annotated by the ClinGen CNV database as implicated in psychiatric or neurodevelopmental disorders (Supplementary Table 10). 14 CNVs with a clinical impact were identified across the samples, but only the 3q29 duplication was present solely in the affected individuals.

**Table 3:** A list of rare discordant CNVs, including: the positions (GRCh38); length; type (*DEL* deletion, *DUP* duplication); the carrier sample ID; their phenotype (*SAD* schizoaffective disorder, *SCZ* schizophrenia); if they are annotated as pathogenic; and the overlapping genes from the BDgene and SZDB databases (full list in Supplementary Tables 8, 9).

Chr	Start	End	Length	Type	Sample	Pheno	Path	BDgene	SZDB
chr3	195940567	197638156	1,697,589	DUP	T17_A	BD	Y	*BDH1*; *DLG1*	20 Genes
chr10	92847856	92849207	1351	DEL	T02_A	SAD			*EXOC6*
chr12	120201498	120204299	2801	DEL	T16_A	BD			
chr19	11261852	11262999	1147	DEL	T01_A1	SCZ			

Somatic CNVs were called using MoChA [53], which examines differences in depth of coverage from phased SNVs (Supplementary Methods). After removing putative false positives on visual inspection of the regions, there does not appear to be evidence for the presence of discordant somatic CNVs in these samples. Repeat expansions were called from the raw sequence data using ExpansionHunter [54] for a collection of 16 repeat expansion disorders (Supplementary Methods, Supplementary Tables 11, 12). Despite some discordances within twin pairs, none of the samples had repeat counts above the specified threshold for any disorder (Supplementary Table 13, Supplementary Fig. 8).

## Discussion

Here we report a WGS study where we assessed de novo post-zygotic variation in blood sample in the affected member of 17 MZ twins discordant for a major psychotic disorder (schizophrenia, schizoaffective disorder, or bipolar disorder). A rigorous filtering strategy identified six rare, deleterious, discordant, protein coding SNVs across four genes, each present in one affected member of the cohort. None of the six missense SNVs appeared in the Schizophrenia Exome Sequencing Meta-Analysis (SCHEMA) database [10], but some variants close by in the amino acid sequence were observed (Supplementary Table 3). In the Bipolar Exome (BipEx) sequencing project [26], one of the S291R variants of *FOXN1* was observed in a bipolar case only. This variant was annotated as “other missense”, a class of variants not found to be enriched in bipolar cases compared to controls. However, all *FOXN1* variants in this study would have been prioritized as “damaging missense” in the SCHEMA analysis. While the *FLOT2* variant was not observed in the BipEx database, it is categorized as “damaging missense”.

We implemented a consensus calling strategy for CNVs and screened for rare CNVs with a known association with psychosis, these being implicated in schizophrenia studies. We identified four rare, discordant CNVs present in affected samples only (Table 3). One such variant was a duplication in the 3q29 region in the affected sample of twin pair T17. While only deletions in this region have been shown to be associated with schizophrenia, this region has also been implicated in autism spectrum disorders and developmental delay. Two of the regions contained genes which had previously shown some relationship to schizophrenia or bipolar disorder (Table 3, Supplementary Tables 8, 9). We investigated an extended list of CNVs reported to have a pathogenic effect in psychiatric or neurodevelopmental disorders (Supplementary Table 10). The previously mentioned 3q29 duplication is the only pathogenic CNV to be found exclusively in affected samples.

A duplication on chromosome 13q12.11 was identified in both samples of twin pair T09. In a discovery association analysis, this CNV was noted to have a protective effect for schizophrenia but was only nominally significant [50]. However, it is worth noting that the affected individual in this twin pair also has the rare, deleterious, discordant missense variant in the *FLOT2* gene (Table 2). *FLOT2* (Flotillin-2) has been shown to be involved in neuronal differentiation [55] and flotillins are known to interact with the NR2A and NR2B subunits of N-methyl-D-aspartate receptors [56].

Given the age profile of some of the twin pairs, it is possible that some of them may have children old enough to have a reliable psychiatric diagnosis. A follow up study including offspring of the twin pairs from this study could allow for the examination of transmission and segregation of the variants we have identified in the next generation. Given the relatively low transmission rates of post-zygotic variants from an MZ twin to their offspring [19], if the variants identified here were found in affected offspring of the MZ twins, this would provide additional support for these variants as disease-causing within that pedigree.

This study has some limitations. First, while the sample size is large relative to other studies of MZ twins, the cohort is not sufficiently powered to statistically evaluate the burden of rare, discordant, protein-coding variants. Second, while we have used the case co-twin design to identify post-zygotic variation, we do not have access to the parental genomes to confirm that our variants are de novo. Parental information would also allow us to examine de novo variation shared between both twins. For example, a shared rare, de novo variant with reduced penetrance could conceivably explain the phenotypic discordance between the twins. Third, the average depth of coverage of the WGS data in this study [30x] may not be sufficient to call variants under a somatic model. Whole exome sequencing, which typically uses a depth of coverage of 90x or higher, has had some success in identifying somatic variants in MZ twins discordant for psychosis [57]. This is supported by the lack of evidence of discordant, somatic CNVs, with all candidate variants being rejected on manual review. Fourth, although we aimed to identify post-zygotic variation occurring early in development, it is possible that de novo variants specific to brain tissue may be present [58], and these may not be observable from blood tissue. Finally, while the samples in this study have undergone a comprehensive diagnostic procedure to ascertain the validity of the phenotypic discordance within each twin pair, the unaffected twin may go on to receive a diagnosis later in life. Future follow up with these individuals from register data may be possible, although many of the twin pairs were originally interviewed after typical age of onset of psychotic disorders.

We have performed the largest MZ twin study for discordant psychotic phenotypes to date. While post-zygotic genomic variations are known to contribute to the discordance in phenotype between MZ twins, other factors such as environmental effects and epigenomic variation can also be driving this discordance. Therefore, many studies of MZ twins also look at methylation differences between MZ twins in addition to genomic variation [59, 60]. In this study, we focused on a broad range of genomic variation, from SNVs (both protein-altering and regulatory), to CNVs and repeat expansions, making use of the full potential of WGS data. This study is important as it contributes novel findings to the current body of literature for variants implicated in psychotic disorders and provides a framework for future studies.

### Supplementary information

Supplementary Material

Supplementary Tables

## Data Availability

The WGS data from the individuals in this study are available from the NIMH Data Archive, collection ID 4231.
