# Supplementary material for: Whole genome sequencing study of identical twins discordant for psychosis
Source: Transl Psychiatry. 2024 Jul 30;14:313. doi: 10.1038/s41398-024-02982-0 (PMC11289105; doi:10.1038/s41398-024-02982-0)
Supplement: Supplementary file 1 — Supplementary Material [file 41398_2024_2982_MOESM1_ESM.docx]

Supplementary Material for

Whole Genome Sequencing Study of Identical Twins Discordant for Psychosis

**Authors**

Cathal Ormond^1^, Niamh M. Ryan^1^, Anna M. Hedman^2^, Tyrone D Cannon^3^, Patrick F. Sullivan^2,4^, Michael Gill^1^, Christina Hultman^2^, Elizabeth A. Heron^1^, Viktoria Johansson^2,5^, Aiden Corvin^1, *^

**Affiliations**

1: Neuropsychiatric Genetics Research Group, Department of Psychiatry, Trinity College Dublin, Ireland

2: Department of Epidemiology and Biostatistics, Karolinska Institutet, Stockholm, Sweden

3: Departments of Psychology and Psychiatry, Yale University School of Medicine, New Haven, CT, USA

4: Departments of Genetics and Psychiatry, University of North Carolina, Chapel Hill, NC, USA

5: Department of Clinical Sciences, Psychiatry Unit, Umeå University, Umeå, Sweden

# Supplementary Methods

## Whole Genome Sequence Data Pre-Processing

Read alignment and pre-processing were performed by Edinburgh Genomics (Clinical Genomics) using the BCBio-Nextgen toolkit, using *HaplotypeCaller* from GATK (version 3.8-0-ge9d806836) to call variants, following the GATK Best Practices workflow (1). All 34 sample were jointly genotyped using the *GenotypeGVCFs* module from GATK using default parameters. After genotyping, a hard filter was applied to remove variants whose depth of coverage was five standard deviations greater than the average depth of coverage across all sites. Variants were then split into SNVs, indels and other variants (multi-nucleotide variants, mixed variants etc.), selecting only those on the standard 23 chromosomes. Variant Quality Score Recalibration (VQSR) was applied to SNVs and indels separately, using default truths sets provided in the GATK resource bundle. A tranche sensitivity threshold of 99.9% was selected for SNVs, and 99.0% for indels. For all other variants, hard filters for indels were applied: QD < 2.0; ReadPosRankSum < -20.0; FS > 200.0; SOR > 10.0. Variants of interest were examined in IGV (2) for visual confirmation of the genotype status.

## Within-Pair Zygosity Confirmation (WGS Data)

The zygosity of both samples within a twin pair was estimated by considering the genotype concordance rates on a set of high-confidence variants. SNVs were retained if they passed the following filters from the jointly genotyped data (from all 34 samples): i) Phred-scaled quality score (QUAL) > 1000.0; ii) depth of coverage (DP) > 100; iii) mapping quality across all reads (MQ) > 5.0; iv) the VQSR log-odds of being a true positive variant (VQSLOD) > 10.0; v) Phred-scaled quality score normalized to read depth (QD) > 5.0. These thresholds were obtained by manually examining the density plots for each of the respective metrics. When filters were applied 6,368,030 SNVs were retained across all samples. Treating one sample within a pair as the “truth” sample, we evaluated the sensitivity and genotype concordance using the *GenotypeConcordance* module form *picard*. The sensitivity measures the proportion of variants in the call set that are present in the truth set, and the genotype concordance measures the proportion of variants with matching genotypes out of those which match a position in the truth set.

## Regulatory Variants and Features

To investigate whether discordant variants across the genome had a predicted regulatory effect, sites were annotated using RegulomeDB (3). A RegulomeDB rank of 2 represents evidence of transcription factor (TF) binding (ChIP-seq data) and a TF motif, as well as evidence of lying under a DNase Footprint and DNase hypersensitive peak. A variant with a rank of 1 requires the same evidence as a rank of 2, as well as evidence suggesting it is within a known eQTL. As RegulomeDB was queried using rsIDs, multi-allelic sites were not split, as it was not possible to determine which allele is regulatory. Given that variants with a regulatory effect can occur in non-coding regions, CADD was used to estimate deleteriousness. The following filters were applied to all discordant variants across the genome: (i) CADD Phred-like scores greater than 20.0; and (ii) RegulomeDB rank of the rsID was 1 or 2, regardless of sub-type; (iii) the allele frequency was <1% or absent in the appropriate population groups in the 1000 Genomes Project and gnomAD databases; and (iv) variants were not observed in any other samples within the cohort.

We compiled a list of features with a known regulatory effect from the Encyclopedia of DNA Elements (4). Specifically, we selected: (i) proximal and distal enhancers; (ii) canonical promoter-like signals; (iii) DNase hypersensitivity sites; (iv) anchors from chromatin loops (ChIA-PET); and (v) transcription factor binding sites with footprints. To this list we added: (vi) brain specific open chromatin regions (5-8); (vii) brain specific enhancers (9); and (viii) proximal promoters of protein-coding transcripts from GENCODE (10). All discordant variants passing QC metrics were subset to these eight regulatory annotation regions. Since the variants were assumed to be post-zygotic, they could be treated as independent events within a twin pair. Due to a low proportion of overlap with data taken from the 1000 Genomes Project, we were unable to evaluate whether LD structure was present within the discordant variants, which may have an effect on the Type I and Type II error rates. A two-tailed t-test at 5% significance was performed using the R software package (version 4.0.2) between samples with or without a diagnosis to evaluate whether there was a significant difference in mean count of discordant variants overlapping a given regulatory annotation.

## CNV Consensus Calling

Germline CNVs were called using a consensus approach (Supplementary Figure 6) based on four tools derived from two classes of calling methods: CNVnator (11) and ERDS (12) (read-depth based callers); LUMPY (13) and Manta (14) (paired-end /split-read based callers). A collapsing strategy was applied to raw CNV calls to eliminate multiple calls which represent the same site, similar to that described in Trost et al. (15). Then, sites that overlapped reciprocally by 50% were merged, first considering within-method callsets (e.g., LUMPY vs Manta) and then between the resulting across-method callsets. Finally, sites for which over 50% of their length comprised of Repeat and Low Complexity Regions (RLCR) were removed. RLCRs are defined in Trost et al. as: (i) assembly gaps, (UCSC “gap” table); (ii) segmental duplications (UCSC “genomicSuperDups” table); (iii) the pseudo-autosomal regions of the sex chromosomes (15).

CNV calls were first screened against a list of 23 rare CNVs (Supplementary Table 6) with significant or nominal association with psychiatric illness (16, 17). Three such CNV regions were called in both samples of five twin pairs (Supplementary Table 7A) but were subsequently rejected as they were identified by one calling algorithm only (LUMPY) and had a low level of support at the breakpoints given the depth of coverage (Supplementary Table 7B).

## Somatic CNV calling

MoChA (18) was applied to the VCF data to investigate the presence of somatic CNVs, based on read-depth information from phased SNVs and indels. The tool was applied with default settings and included a list of regions to exclude for WGS data. Any two CNV regions that overlapped reciprocally by at least 50% were denoted as the same. As above, deletions or duplications for which at least 50% of their length comprised of RLCR were removed. Any CNV call that was identified in the germline call set above was also removed. Finally, CNV calls present in both samples of a twin pair were removed. The read-depth profile for the regions of interest were plotted for carriers and their co-twin to confirm that the calls were discordant.

## Multi-nucleotide repeat calling

Multi-nucleotide repeat expansions were called from the BAM files using ExpansionHunter (19). Variant regions for a collection of 15 repeat disorders (20) were taken from the variant catalogue supplied with the tool. To this list was added the GGCCCC repeat in the C9ORF72 gene, known to be causal for certain forms of frontotemporal dementia (FTD) and amyotrophic lateral sclerosis (ALS) (21). While this tool was developed for PCR-free WGS data, this was accounted for by examining the coverage for each repeat region to ensure it was not low. As the average depth of coverage was greater than 14x for all multi-nucleotide repeat regions across all samples (Supplementary Table 12), the data were deemed of sufficient quality.

## Gene Databases

Genes affected by variants of interest were screened against several publicly available databases. The BDGene database (22) lists genes and the studies that show positive, negative or suggestive evidence of association with bipolar disorder. Genes with at least one positive study reported in BDGene were considered. The Schizophrenia Database (SZDB) (23) compiles various studies on schizophrenia, including GWAS, linkage, differential expression, differential methylation etc. Genes that had any level of evidence of association with schizophrenia from the SZDB database were considered.

## Web Resources

- FastQC: http://www.bioinformatics.babraham.ac.uk/projects/fastqc/
- Picard tools: https://broadinstitute.github.io/picard/
- Karlinska Institutet Biobank: http://ki.se/forskning/ki-biobank

# Supplementary Tables

Supplementary Table 1: Sequencing details for all 34 samples, including the average depth of coverage, the number of raw, high quality, twin-pair private, and discordant SNVs/Indels, and the number of raw and discordant CNV calls.

| **Sample** | **Coverage** | **SNV/Indels** | | | | **CNV** | |
| --- | --- | --- | --- | --- | --- | --- | --- |
|  |  | **Raw** | **HighQual** | **Private** | **Disc.** | **Raw** | **Disc.** |
| T01_A1 | 37.7 | 5,135,840 | 4,559,707 | 155,294 | 46,802 | 13,328 | 66 |
| T01_A2 | 39.0 | 5,132,951 | 4,554,292 | 155,294 | 48,050 | 13,276 | 72 |
| T02_A | 37.3 | 5,086,838 | 4,500,614 | 164,627 | 42,299 | 12,050 | 66 |
| T02_U | 38.0 | 5,091,627 | 4,502,207 | 164,627 | 44,221 | 12,208 | 74 |
| T03_A | 36.1 | 5,124,533 | 4,560,664 | 158,000 | 43,669 | 12,434 | 53 |
| T03_U | 36.4 | 5,122,960 | 4,551,756 | 158,000 | 43,609 | 12,515 | 74 |
| T05_A1 | 36.0 | 5,065,741 | 4,473,264 | 350,387 | 41,134 | 12,794 | 88 |
| T05_A2 | 35.5 | 5,057,900 | 4,463,092 | 350,387 | 41,321 | 12,567 | 66 |
| T06_A | 36.7 | 5,134,960 | 4,578,265 | 165,262 | 45,466 | 12,717 | 65 |
| T06_U | 37.2 | 5,140,021 | 4,582,792 | 165,262 | 46,195 | 12,854 | 73 |
| T07_A | 37.7 | 5,124,989 | 4,564,116 | 155,563 | 45,180 | 12,078 | 69 |
| T07_U | 38.9 | 5,109,831 | 4,559,035 | 155,563 | 44,197 | 12,084 | 90 |
| T08_A | 38.6 | 5,080,887 | 4,517,086 | 155,207 | 43,370 | 12,486 | 60 |
| T08_U | 38.1 | 5,060,584 | 4,494,792 | 155,207 | 43,084 | 13,176 | 91 |
| T09_A | 34.6 | 5,123,584 | 4,562,983 | 154,868 | 44,628 | 12,003 | 78 |
| T09_U | 37.4 | 5,124,871 | 4,571,913 | 154,868 | 44,983 | 12,330 | 78 |
| T10_A | 37.1 | 4,975,315 | 4,388,183 | 151,053 | 45,856 | 12,193 | 67 |
| T10_U | 37.5 | 5,001,430 | 4,414,223 | 151,053 | 45,320 | 12,862 | 66 |
| T11_A | 32.7 | 5,129,037 | 4,556,925 | 161,090 | 43,156 | 12,179 | 48 |
| T11_U | 35.4 | 5,094,592 | 4,504,299 | 161,090 | 43,162 | 11,576 | 57 |
| T12_A | 37.8 | 5,105,383 | 4,536,345 | 151,637 | 44,258 | 12,492 | 84 |
| T12_U | 35.7 | 5,055,787 | 4,468,107 | 151,637 | 44,269 | 11,245 | 52 |
| T13_A1 | 35.9 | 5,086,950 | 4,518,169 | 149,161 | 42,268 | 13,351 | 100 |
| T13_A2 | 35.5 | 5,042,295 | 4,449,752 | 149,161 | 42,771 | 12,435 | 70 |
| T14_A | 35.6 | 5,069,211 | 4,496,725 | 150,611 | 41,909 | 12,892 | 93 |
| T14_U | 34.0 | 5,059,881 | 4,458,123 | 150,611 | 43,214 | 13,505 | 53 |
| T15_A1 | 37.6 | 5,092,231 | 4,514,351 | 146,164 | 45,031 | 12,073 | 74 |
| T15_A2 | 36.1 | 5,099,361 | 4,522,178 | 146,164 | 47,466 | 12,110 | 68 |
| T16_A | 35.8 | 5,091,049 | 4,513,939 | 153,184 | 44,729 | 12,129 | 55 |
| T16_U | 36.9 | 5,119,736 | 4,547,162 | 153,184 | 44,794 | 12,067 | 83 |
| T17_A | 36.2 | 5,136,974 | 4,578,218 | 153,518 | 43,595 | 12,978 | 74 |
| T17_U | 37.0 | 5,133,944 | 4,568,041 | 153,518 | 44,627 | 12,433 | 62 |
| T18_A1 | 35.4 | 5,050,158 | 4,483,907 | 159,325 | 46,048 | 12,298 | 75 |
| T18_A2 | 38.5 | 5,094,623 | 4,541,999 | 159,325 | 45,707 | 13,509 | 86 |

Supplementary Table 2: Within-pair concordance metrics on a set of high-confidence SNVs for the 17 MZ twin pairs.

| **Twin Pair** | **Truth Sample** | **Call Sample** | **Sensitivity** | **Genotype Concordance** |
| --- | --- | --- | --- | --- |
| T01 | T01_A1 | T01_A2 | 99.8994% | 99.9998% |
|  | T01_A2 | T01_A1 | 99.9058% | 99.9998% |
| T02 | T02_A | T02_U | 99.8854% | 99.9997% |
|  | T02_U | T02_A | 99.8908% | 99.9997% |
| T03 | T03_A | T03_U | 99.8925% | 99.9997% |
|  | T03_U | T03_A | 99.8910% | 99.9997% |
| T05 | T05_A1 | T05_A2 | 99.8848% | 99.9997% |
|  | T05_A2 | T05_A1 | 99.8904% | 99.9997% |
| T06 | T06_A | T06_U | 99.8949% | 99.9997% |
|  | T06_U | T06_A | 99.8955% | 99.9997% |
| T07 | T07_A | T07_U | 99.8924% | 99.9997% |
|  | T07_U | T07_A | 99.8912% | 99.9997% |
| T08 | T08_A | T08_U | 99.8881% | 99.9997% |
|  | T08_U | T08_A | 99.8949% | 99.9997% |
| T09 | T09_A | T09_U | 99.8931% | 99.9997% |
|  | T09_U | T09_A | 99.8906% | 99.9997% |
| T10 | T10_A | T10_U | 99.8944% | 99.9998% |
|  | T10_U | T10_A | 99.8902% | 99.9998% |
| T11 | T11_A | T11_U | 99.8686% | 99.9997% |
|  | T11_U | T11_A | 99.8867% | 99.9997% |
| T12 | T12_A | T12_U | 99.8920% | 99.9998% |
|  | T12_U | T12_A | 99.8981% | 99.9998% |
| T13 | T13_A1 | T13_A2 | 99.8739% | 99.9997% |
|  | T13_A2 | T13_A1 | 99.8952% | 99.9997% |
| T14 | T14_A | T14_U | 99.8709% | 99.9997% |
|  | T14_U | T14_A | 99.8892% | 99.9997% |
| T15 | T15_A1 | T15_A2 | 99.8871% | 99.9997% |
|  | T15_A2 | T15_A1 | 99.8936% | 99.9997% |
| T16 | T16_A | T16_U | 99.8986% | 99.9997% |
|  | T16_U | T16_A | 99.8881% | 99.9997% |
| T17 | T17_A | T17_U | 99.8953% | 99.9997% |
|  | T17_U | T17_A | 99.8950% | 99.9997% |
| T18 | T18_A1 | T18_A2 | 99.9005% | 99.9998% |
|  | T18_A2 | T18_A1 | 99.8876% | 99.9998% |

Supplementary Table 3: The nine rare, discordant, predicted-deleterious SNVs present in affected twin samples, and evidence from the Schizophrenia Exome Sequencing Meta-Analysis (SCHEMA) consortium (24) of rare variants affecting amino acids ± 10 positions in the in the amino acid sequence of the gene. Annotated is the functional consequence of the variant, and the number of times the allele was observed across cases (total of 48,496 alleles) and controls (total of 194,644 alleles).

| **Chr** | **Pos** | **Gene** | **HGVS_P** | **SCHEMA** | **Consequence** | **Cases** | **Controls** |
| --- | --- | --- | --- | --- | --- | --- | --- |
| chr9 | 96932219 | *NUTM2G* | p.Pro172Ser | - | - | - | - |
| chr17 | 28530789 | *FOXN1* | p.Ser291Arg | - | - | - | - |
| chr17 | 28530791 |  | p.Ser291Arg | - | - | - | - |
| chr17 | 28530802 |  | p.Ser295Thr | - | - | - | - |
| chr17 | 28881251 | *FLOT2* | p.Ala347Thr | p.Ala338Pro | Missense | 1 | 0 |
|  |  |  |  | p.Tyr351His | Missense | 1 | 1 |
| chr22 | 44592351 | *KRTAP10-6* | p.Pro45Arg | p.Ser54Asn | Missense | 0 | 1 |
|  |  |  |  | p.Pro44Ala | Missense | 0 | 1 |
|  |  |  |  | p.Asp36Ala | Missense | 184 | 291 |

Supplementary Table 4: Rare, discordant, deleterious variants with a predicted regulatory effect. Each variant is annotated with: genomic positions (GRCh38); rsIDs; the reference and alternative alleles of the variant; the sample carrying the variant; the phenotype of the sample; the CADD (Phred) score; the RegulomeDB score; the minor allele frequency from gnomAD.

| **Chr** | **Pos** | **rsID** | **Ref** | **Alt** | **Sample** | **Pheno** | **CADD** | **Rank** | **MAF** |
| --- | --- | --- | --- | --- | --- | --- | --- | --- | --- |
| chr16 | 49858208 | rs1022231788 | G | T | T09_U | None | 21.8 | 2b | 0.000719 |

Supplementary Table 5: Results from the paired, two-sided Wilcoxon signed-rank test to evaluate the enrichment of discordant variants in various regulatory features within twin pairs.

| **Regulatory Feature** | **Description** | ***p*** |
| --- | --- | --- |
|  |  |  |
| pc_promoter500 | Promoters of protein-coding transcripts | 0.85 |
| reg_anchor | Anchors from chromatin loops | 0.58 |
| reg_dhs | DNase hypersensitivity sites | 0.33 |
| reg_enhancer | Proximal/distal enhancers | 0.93 |
| reg_enhancerbrain | Brain-specific enhancers | 0.85 |
| reg_openbrain | Brain-specific open chromatin regions | 0.82 |
| reg_promoter | Promoter-like signals | 0.41 |
| reg_tfbs | Transcription factor binding sites | 0.48 |

Supplementary Table 6: A list of 23 rare CNVs from 19 unique regions, with a known association with schizophrenia, taken from (16, 17).

| **Chr** | **Start** | **End** | **Locus (gene)** | **Type** | **OR (95% CI)** | **Notes** |
| --- | --- | --- | --- | --- | --- | --- |
| chr1 | 147068456 | 147917873 | 1q21.1 | DUP | 3.45 (1.92 - 6.20) |  |
|  |  |  |  | DEL | 8.35 (4.65 - 14.99) |  |
| chr2 | 49922862 | 51032862 | 2p16.3 (*NRXN1*) | DEL | 9.01 (4.44 - 18.29) |  |
| chr3 | 196003129 | 197613129 | 3q29 | DEL | 57.65 (7.58 - 438.44) |  |
| chr7 | 65373855 | 65401085 | 7q.11.21 (*ZNF92*) | DEL | 0.66 (0.52 - 0.84) | Nominal significance. |
|  |  |  |  | DUP | 0.66 (0.52 - 0.84) | Nominal significance. |
| chr7 | 73325996 | 74725666 | 7q11.23 | DUP | 11.35 (2.58 - 49.93) |  |
| chr7 | 158660506 | 159179546 | 7p.36.3 (*VIPR2*; *WDR60*) | DEL | 3.5 (1.3 - 9.0) | Nominal significance. |
|  |  |  |  | DUP | 3.5 (1.3 - 9.0) | Nominal significance. |
| chr8 | 99013266 | 99877580 | 8q.22.2 (*VPS13B*) | DEL | 14.5 (1.7 - 122.2) | Nominal significance. |
| chr9 | 841690 | 969090 | 9p.24.3 (*DMRT1*) | DEL | 12.4 (1.6 - 98.1) | Nominal significance. |
|  |  |  |  | DUP | 12.4 (1.6 - 98.1) | Nominal significance. |
| chr13 | 19837453 | 19863633 | 13q.12.11 (*ZMYM5*) | DUP | 0.36 (0.19 - 0.67) | Nominal significance. |
| chr15 | 22783067 | 23073067 | 15q11.2 | DEL | 2.15 (1.71 - 2.68) |  |
| chr15 | 24574853 | 28184854 | 15q11 | DUP | 13.20 (3.72 - 46.77) | Angelman/Prader-Wili syndromes. |
| chr15 | 30837797 | 32187799 | 15q13.3 | DEL | 7.52 (3.98 - 14.19) |  |
| chr16 | 15416143 | 16206143 | 16p13.11 | DUP | 2.3 (1.57 - 3.36) |  |
| chr16 | 21938679 | 22418679 | 16p12.1 | DEL | 3.3 (1.61 - 7.05) |  |
| chr16 | 28808679 | 29038679 | 16p11.2, distal | DEL | 20.6 (2.6 - 162.2) |  |
| chr16 | 29628679 | 30188679 | 16p11.2, proximal | DUP | 11.52 (6.86 - 19.34) |  |
| chr22 | 19032487 | 20272477 | 22q11.2 | DEL | INF (28.27 - INF) |  |
| chrX | 149712024 | 149717267 | Xq28 (*MAGEA11*) | DUP | 0.35 (0.18 - 0.68) | Nominal significance. |
| chrX | 154918531 | 155342497 | Xq28 | DUP | 8.9 (2.0 - 39.9) | Nominal significance. |

Supplementary Table 7 A.& B.: CNVs with known psychiatric association identified in the cohort. In both tables (A) and (B), the start and end points are given for the GRCh38 reference genome. (A) A breakdown of which of the four callers identified the CNV (C – CNVnator; E – ERDS; L – LUMPY; M – Manta). (B) For the three CNVs identified by LUMPY alone, the number of paired end (PE) reads that support the event, the read depth at the start of the CNV (DP_S) and the read depth at the end of the CNV (DP_E).

(A)

| **Chr** | **Start** | **End** | **Name** | **Twin** | **Affected** | | | | **Unaffected** | | | |
| --- | --- | --- | --- | --- | --- | --- | --- | --- | --- | --- | --- | --- |
|  |  |  |  |  | **C** | **E** | **L** | **M** | **C** | **E** | **L** | **M** |
| chr3 | 195945160 | 197641345 | 3q29_DEL | T06 |  |  | × |  |  |  | × |  |
| chr16 | 21776296 | 22592576 | 16p12.1_DEL | T08 |  |  | × |  |  |  | × |  |
| chr13 | 19841000 | 19874999 | 13q12.11_DUP | T09 | × | × | × | × | × | × | × | × |
| chr15 | 22776711 | 28851112 | 15q11.2_DEL | T11 |  |  | × |  |  |  | × |  |
| chr15 | 22775347 | 28851109 | 15q11.2_DEL | T12 |  |  | × |  |  |  | × |  |
| chr15 | 22776709 | 28851099 | 15q11.2_DEL | T17 |  |  | × |  |  |  | × |  |

(B)

| **Chr** | **Start** | **End** | **Name** | **Twin** | **Affected** | | | | **Unaffected** | | |
| --- | --- | --- | --- | --- | --- | --- | --- | --- | --- | --- | --- |
|  |  |  |  |  | **PE** | **DP_S** | **DP_E** | **PE** | | **DP_S** | **DP_E** |
| chr3 | 195945160 | 197641345 | 3q29_DEL | T06 | 4 | 89 | 73 | 4 | | 131 | 61 |
| chr16 | 21776296 | 22592576 | 16p12.1_DEL | T08 | 7 | 51 | 59 | 4 | | 57 | 68 |
| chr15 | 22776711 | 28851112 | 15q11.2_DEL | T11 | 10 | 53 | 21 | 11 | | 40 | 33 |
| chr15 | 22775347 | 28851109 | 15q11.2_DEL | T12 | 4 | 34 | 35 | 9 | | 34 | 30 |
| chr15 | 22657499 | 28851699 | 15q11.2_DEL | T17 | 4 | 25 | 28 | 4 | | 41 | 32 |

Supplementary Table 8: Rare, discordant CNVs which affect genes present in the SZDB database (25). A gene is reported if it has an association with schizophrenia as identified by known CNV regions, differential methylation, differential gene expression, rare-variant exome sequencing burden analysis, if it is expressed in brain tissues, or if the gene ontology is related to brain/neuron function.

[Supplied separately]

Supplementary Table 9: Rare, discordant CNVs which overlap genes present in the BDgene database (22). The genes are annotated with the number of studies that showed positive/negative/suggestive evidence of association with bipolar disorder, as well as if the genes were implicated in studies of schizophrenia (SCZ) and major depressive disorder (MDD).

| **CHR** | **START** | **END** | **Location** | **Gene** | **Studies** | | | **SCZ** | **MDD** |
| --- | --- | --- | --- | --- | --- | --- | --- | --- | --- |
|  |  |  |  |  | **Pos** | **Neg** | **Trend** |  |  |
| chr3 | 195940567 | 197638156 | 3q29 | *BDH1* | 1 | 1 | 0 | NO | NO |
|  |  |  |  | *DLG1* | 1 | 1 | 0 | YES | NO |
| chr11 | 19858200 | 19861999 | 11p15.1 | *NAV2* | 1 | 0 | 0 | NO | YES |

Supplementary Table 10: A list of CNVs with a predicted pathogenic effect in ClinGen, including the positions (GRCh38), length, the associated phenotypes, the CNV type, the number of affected (A) and unaffected (U) samples who carried a discordant variant (Disc), and the number of samples who carried the variant (ALL). ADHD: Attention Deficit/Hyperactivity Disorder; ASD: Autism Spectrum Disorder; DD: Developmental Delay; GDD: Global Developmental Delay; ID: Intellectual Disability; DEL: deletion; DUP: duplication.

| **Chr** | **Start** | **End** | **Length** | **Phenotypes** | **Type** | **Disc** | | **All** |
| --- | --- | --- | --- | --- | --- | --- | --- | --- |
|  |  |  |  |  |  | **A** | **U** |  |
| chr1 | 143,707,655 | 148,368,205 | 4,660,550 | ASD; DD; GDD; ADHD; Seizures; ID | DEL | 0 | 1 | 3 |
| chr2 | 95,974,322 | 97,579,728 | 1,605,406 | DD; ASD; ID; Seizures | DEL | 4 | 1 | 7 |
| chr3 | 1,880,400 | 6,681,218 | 4,800,818 | DD; GDD | DEL | 0 | 1 | 9 |
|  |  |  |  |  | DUP | 1 | 2 | 5 |
| chr3 | 195,940,567 | 197,638,156 | 1,697,589 | ASD; DD; GDD; Seizures | DUP | 1 | 0 | 1 |
| chr5 | 141,631,941 | 142,199,762 | 567,821 | DD; GDD | DUP | 1 | 0 | 21 |
|  |  |  |  |  | DEL | 1 | 0 | 27 |
| chr7 | 66,392,490 | 76,087,266 | 9,694,776 | DD; ID | DEL | 1 | 0 | 7 |
|  |  |  |  |  | DUP | 1 | 1 | 20 |
| chr12 | 676,066 | 1,623,296 | 947,230 | DD; GDD; Seizures | DEL | 0 | 1 | 1 |
| chr12 | 120,442,095 | 121,624,256 | 1,182,161 | GDD, Seizures | DUP | 2 | 1 | 25 |
| chr16 | 21,935,407 | 29,107,962 | 7,172,555 | ID; DD; ASD | DUP | 1 | 1 | 12 |
| chr16 | 28,470,489 | 29,397,846 | 927,357 | DD; Seizures; ASD; GDD; ID | DUP | 1 | 0 | 11 |
| chr17 | 36,271,243 | 37,995,798 | 1,724,555 | DD; Seizures; GDD; ID; ASD; Anorexia | DEL | 2 | 1 | 11 |
| chrX | 66,699,870 | 84,875,177 | 18,175,307 | DD | DUP | 2 | 1 | 5 |
| chrX | 69,751,300 | 78,489,674 | 8,738,374 | DD | DUP | 1 | 0 | 9 |
|  |  |  |  |  | DEL | 1 | 0 | 7 |
| chrX | 94,704,302 | 98,524,458 | 3,820,156 | Seizures | DUP | 1 | 0 | 3 |

Supplementary Table 11: List of disorders associated with a multi-nucleotide repeat expansion, and their pathogenic repeat count threshold, taken from (21, 26).

| **Disorder** | **Alias** | **Location** | **Repeat** | **Threshold** |
| --- | --- | --- | --- | --- |
| Fragile X Syndrome | FRAXA | Xq27.3 | CGC | 200 |
| Fragile X–Associated Tremor Ataxia Syndrome | FXTAS | Xq27.3 | CGG | 60 |
| Fragile XE Syndrome | FRAXE | Xq28 | CCG | 200 |
| Friedreich ataxia | FRDA | 9q21.11 | GAA | 200 |
| Myotonic Dystrophy 1 | DM1 | 19q13.32 | CTG | 50 |
| Myotonic Dystrophy 2 | DM2 | 3q21.3 | CCTG | 75 |
| Spinobulbar Muscular Atrophy | SBMA | Xq11-12 | CAG | 38 |
| Huntington Disease | HD | 4p16.3 | CAG | 36 |
| Spinocerebellar Ataxia 1 | SCA1 | 6p22.3 | CAG | 39 |
| Spinocerebellar Ataxia 2 | SCA2 | 12q24.12 | CAG | 32 |
| Spinocerebellar Ataxia 3 | SCA3 | 14q32.12 | CAG | 61 |
| Spinocerebellar Ataxia 6 | SCA6 | 19p13.13 | CAG | 20 |
| Spinocerebellar Ataxia 7 | SCA7 | 3p14.1 | CAG | 37 |
| Spinocerebellar Ataxia 17 | SCA17 | 6q27 | CAG | 47 |
| Dentatorubropallidoluysian atrophy | DRPLA | 12p13.31 | CAG | 49 |
| Amyotrophic Lateral Sclerosis | ALS | 9p21.2 | GGGGCC | 30 |

Supplementary Table 12: Average depth of coverage in the MZ twin cohort across repeat regions for multi-nucleotide repeat expansions.

[Supplied separately]

Supplementary Table 13: Repeat counts (maximum across both alleles) for the 16 repeat expansions associated with expansion disorders, identified by *ExpansionHunter* within the MZ twin cohort (19).

[Supplied separately]

# Supplementary Figures

Supplementary Figure 1: Flow chart of the CNV consensus calling algorithm, with the paired-end/split-read callers highlighted in red, and the read-depth callers highlighted in green. RLCR: Repeat/low complexity regions;


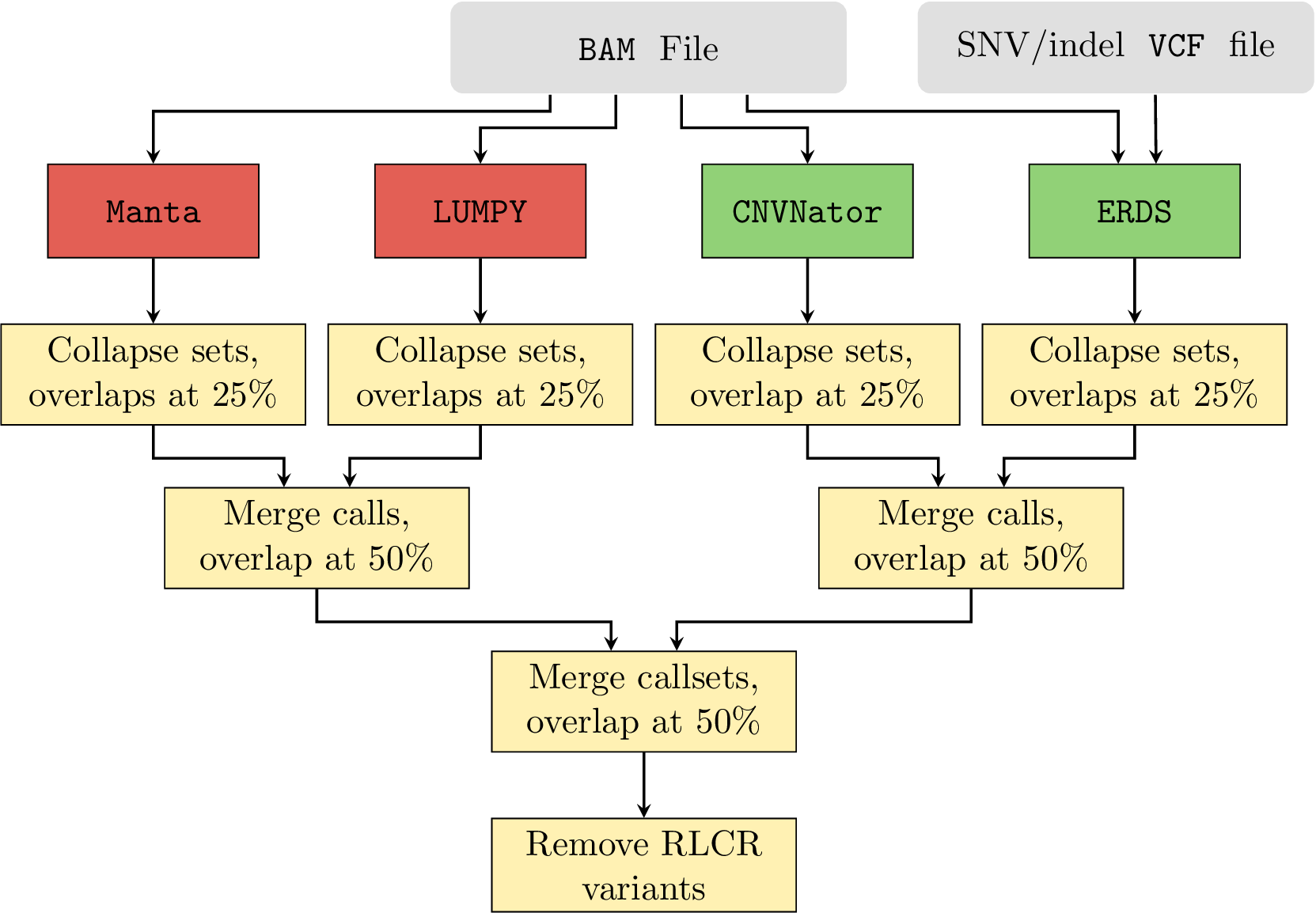


Supplementary Figure 2: Flow chart of the within-twin pair merging, and common variant removal.


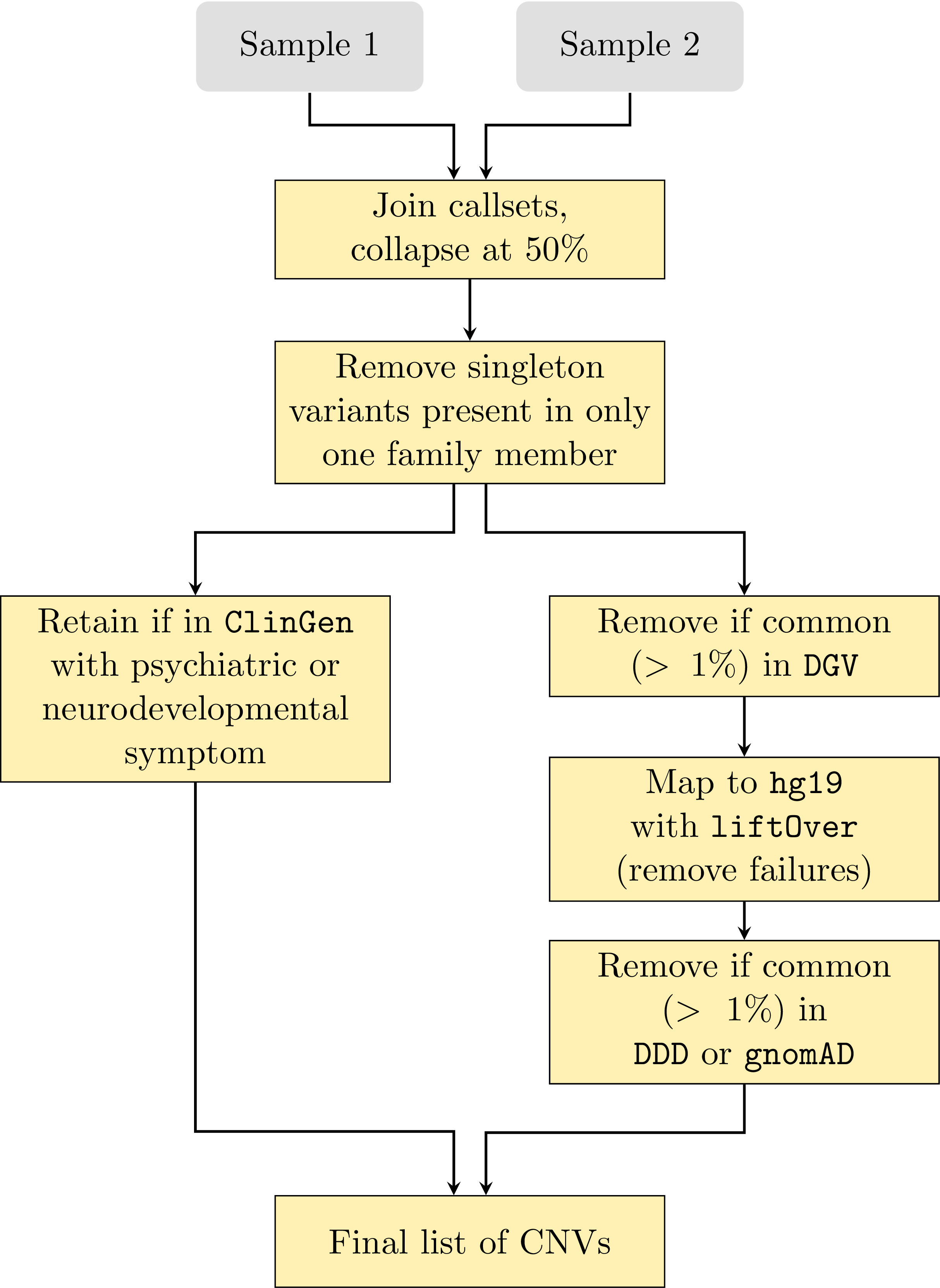


Supplementary Figure 3: A plot of the first two principal components of the MZ twins and a background population from the 1000 Genome Project, re-generated from the output of *peddy* (27). AFR: African; AMR: American; EAS: East Asian; EUR: European; MZT: monozygotic twins; SAS: South Asian.


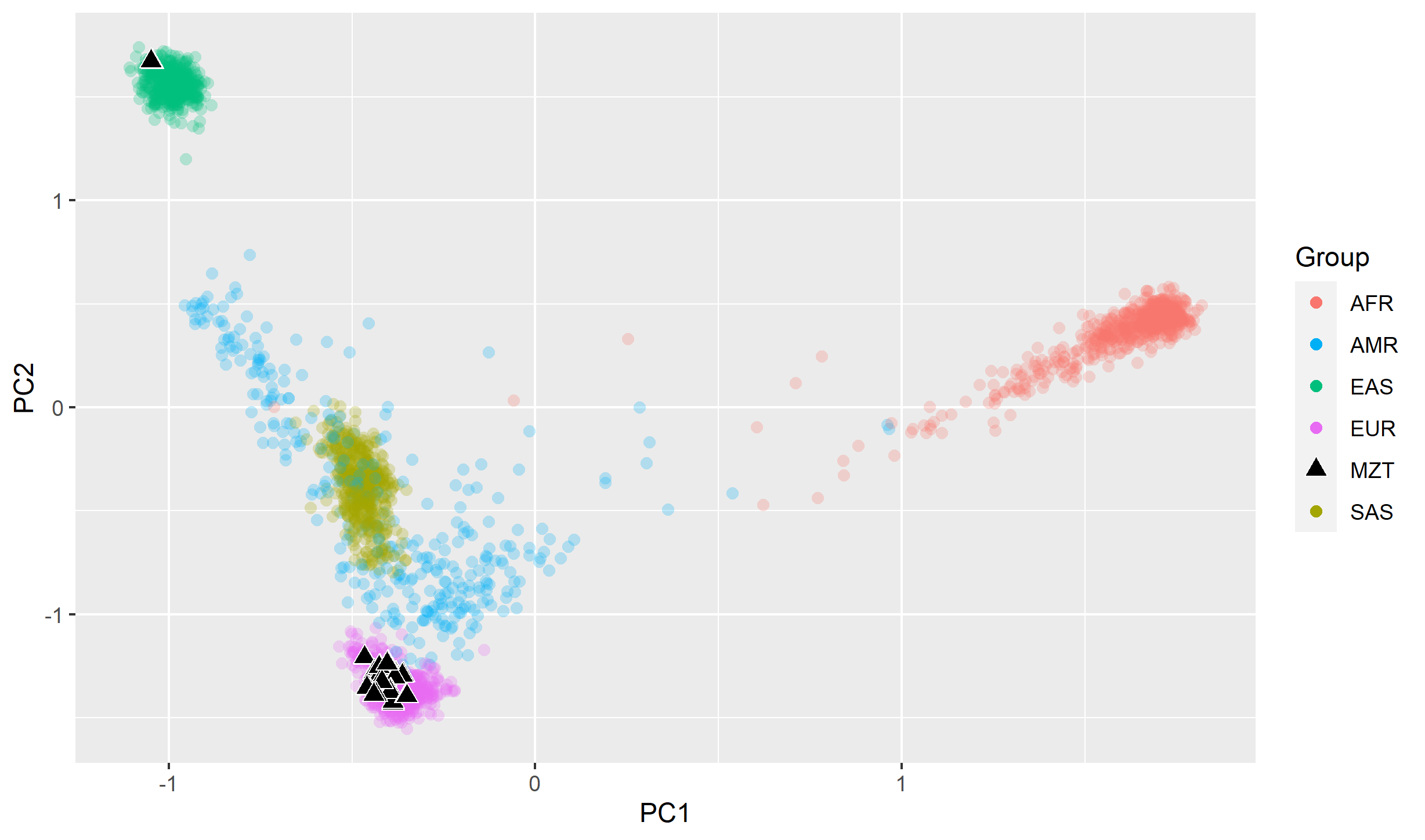


Supplementary Figure 4: A plot of the first two principal components of the MZ twins and a background population from the 1000 Genome Project, centered on the European sub-section. AMR: American; EUR: European; MZT: monozygotic twins.


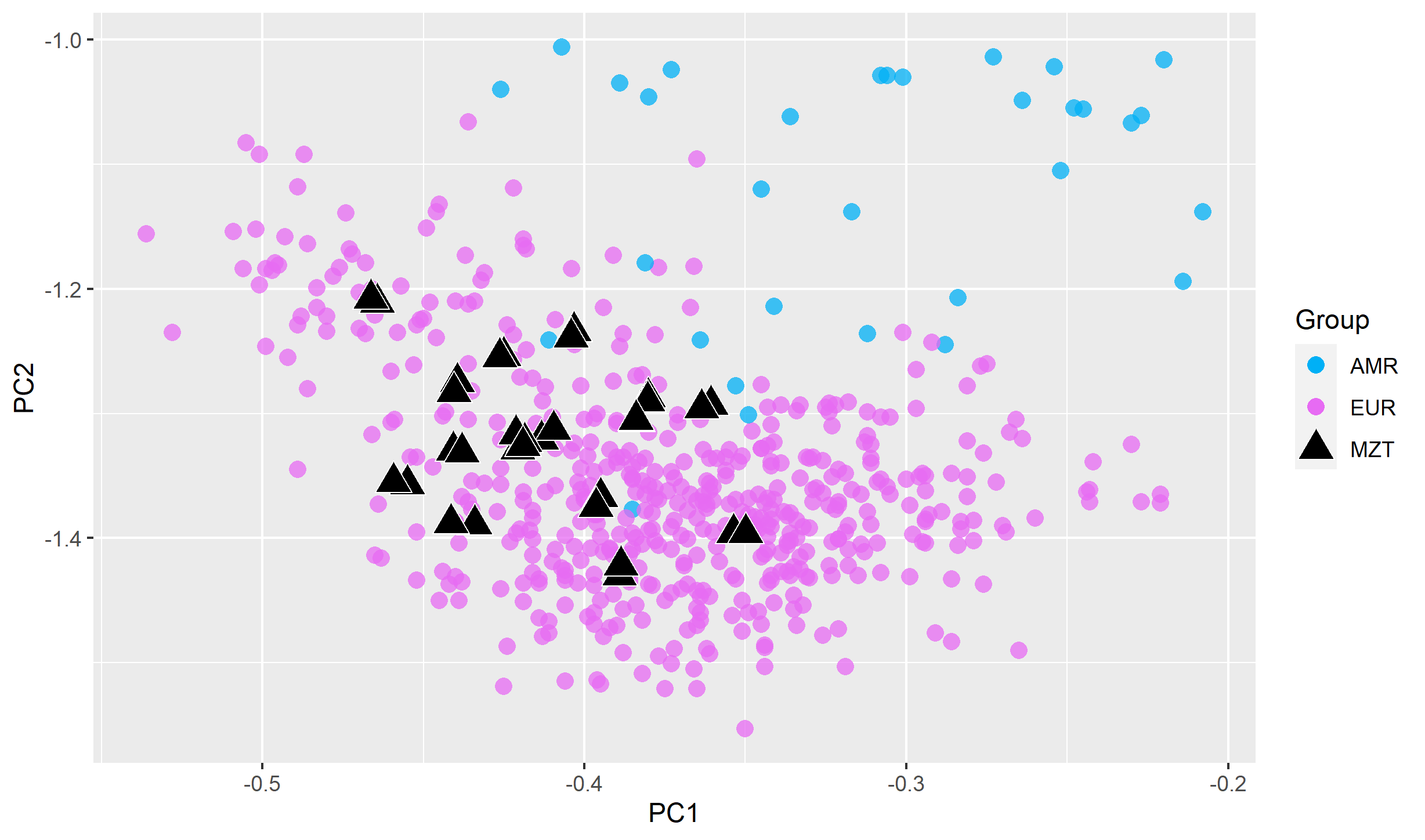


Supplementary Figure 5: A plot of the first two principal components of the MZ twins and a background population from the 1000 Genome Project, centered on the East Asian sub-section. EAS: East Asian; MZT: monozygotic twins.


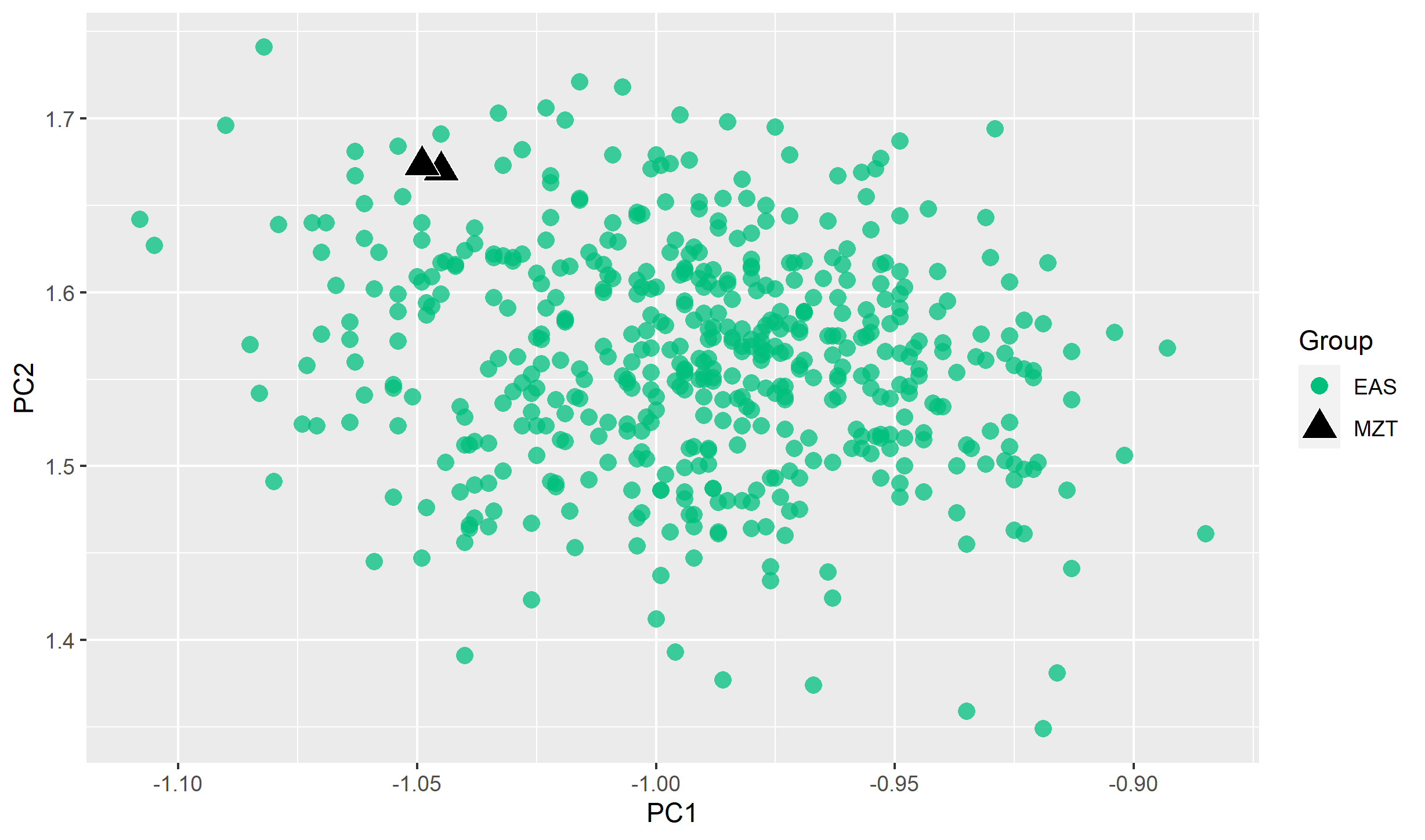


Supplementary Figure 6: IGV plot (2) of the *bamout* output of *HaplotypeCaller* for sample T07_A, showing three SNVs in *FOXN1* (highlighted in yellow) arising from re-constructed reads due to local re-alignment around indels. Reconstructed reads are displayed in color, regular reads are shown in grey.


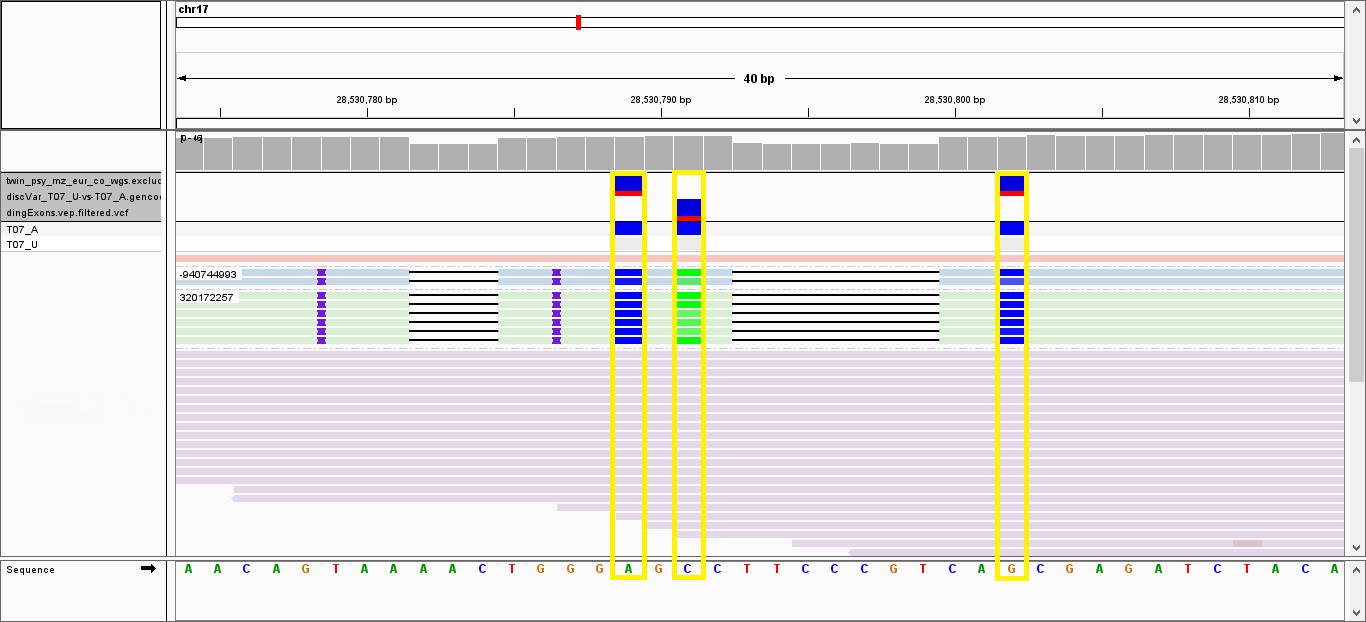


Supplementary Figure 7: Boxplots of the counts of discordant variants within each of the eight regulatory annotation sets. The affected samples are displayed in red, and the unaffected samples are displayed in blue. The p-value from a paired Wilcoxon test is displayed above each pair of boxplots. The selected regulatory features are described in Supplementary Table 5.


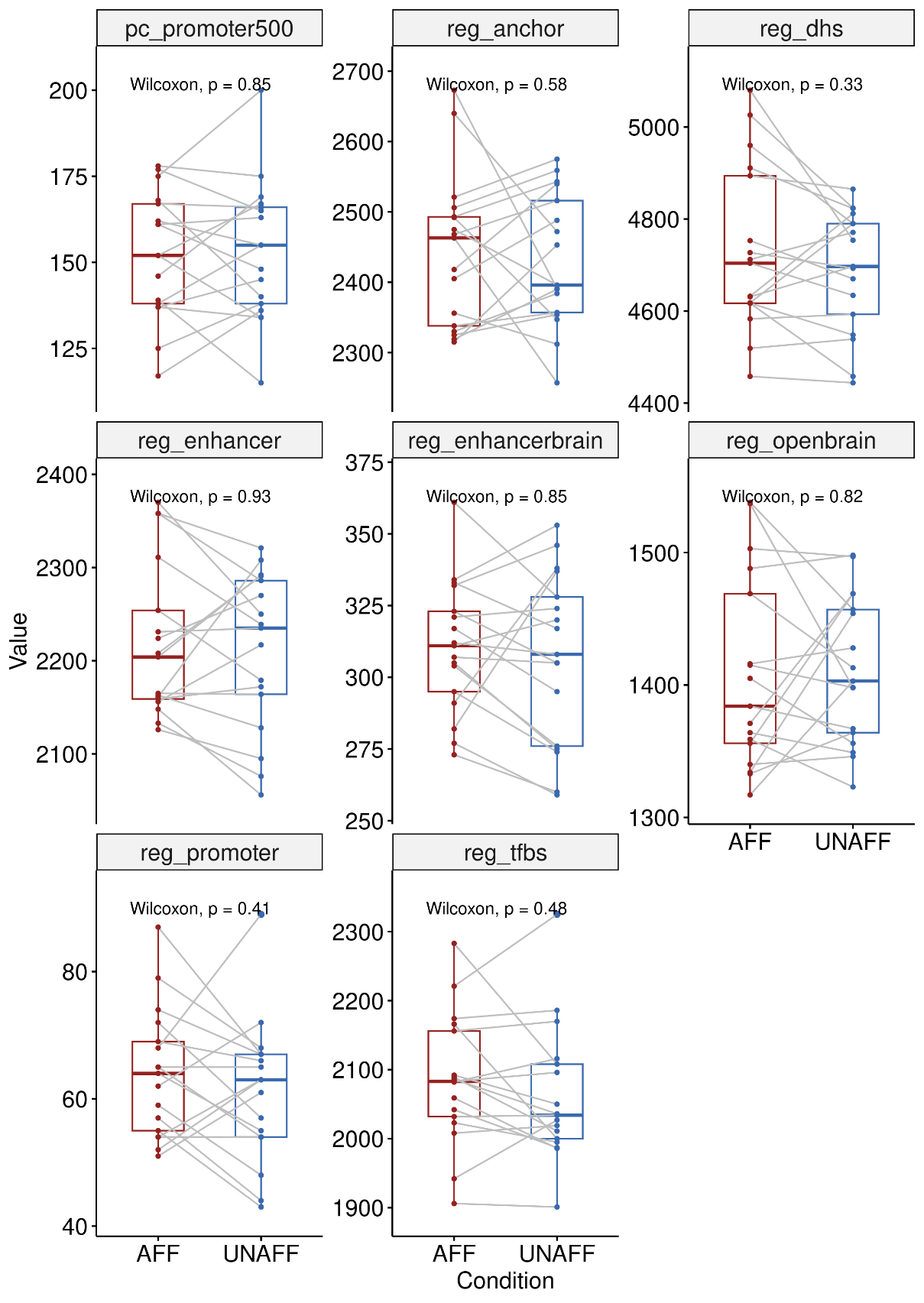


Supplementary Figure 8: Read depth (RD) and estimated phased b-allele frequency (pBAF) plots for two somatic CNVs identified by *MoChA* (18) as discordant within twin pairs. Called CNVs are colored in red/blue. (A) In twin pair T07, the read depth profile is similar, in both samples across the region identified, but the CNV was not called in sample T07_U. (B) In twin pair T08, CNV was called in both samples, but the breakpoints are not the same, despite a similar read-depth profile.

(A)


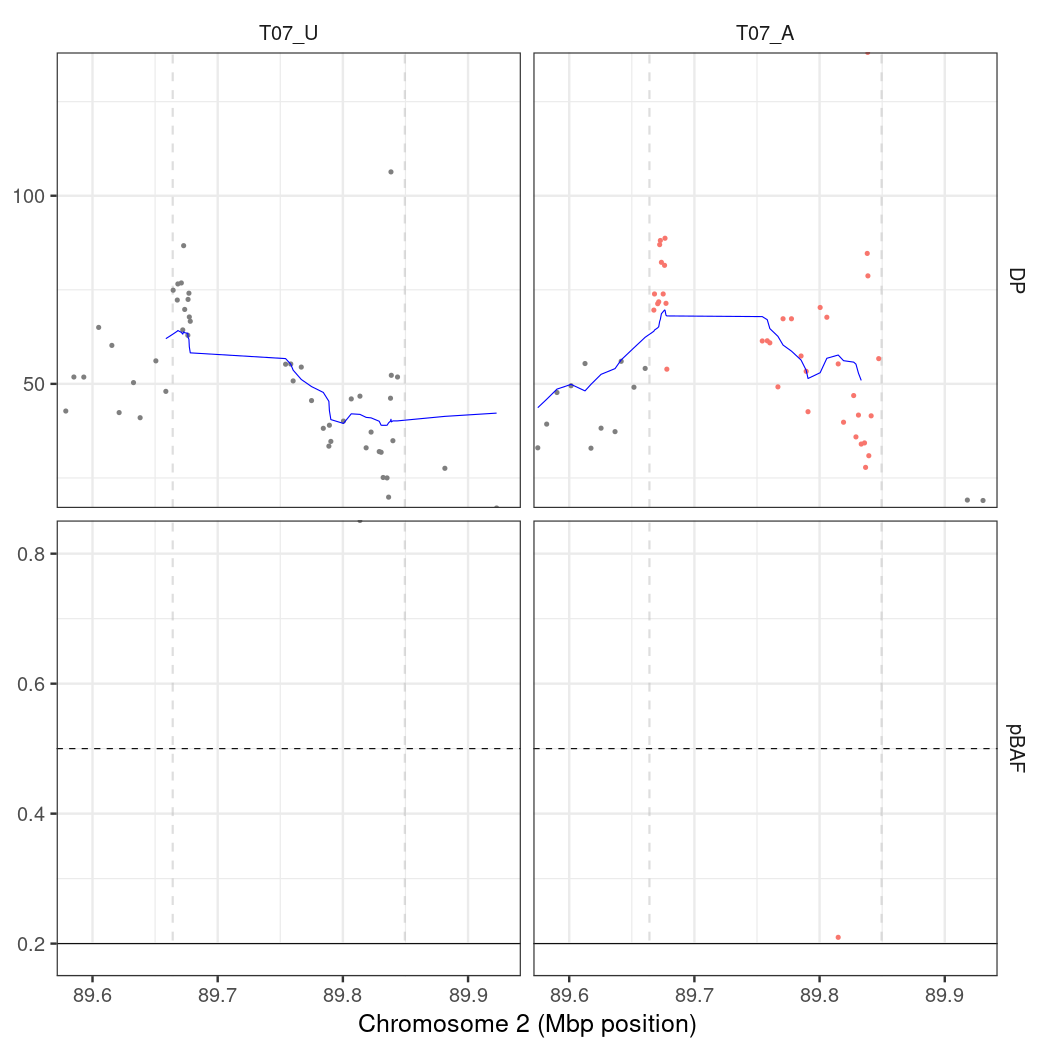


(B)


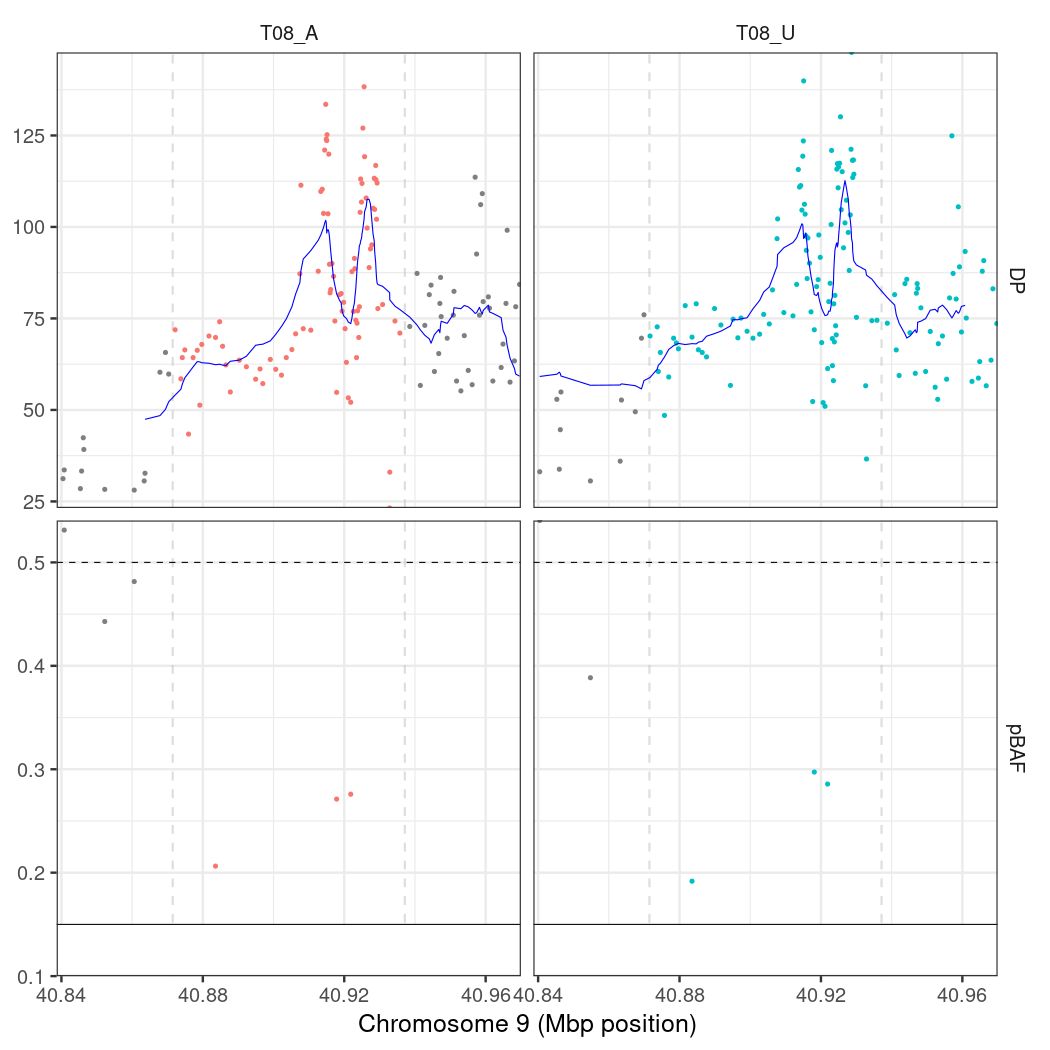


# References

1. Van der Auwera GA, Carneiro MO, Hartl C, Poplin R, Del Angel G, Levy-Moonshine A, et al. (2013): From FastQ data to high confidence variant calls: the Genome Analysis Toolkit best practices pipeline. *Current protocols in bioinformatics*. 43:11.10.11-33.

2. Robinson JT, Thorvaldsdóttir H, Wenger AM, Zehir A, Mesirov JP (2017): Variant Review with the Integrative Genomics Viewer. *Cancer Res*. 77:e31-e34.

3. Boyle AP, Hong EL, Hariharan M, Cheng Y, Schaub MA, Kasowski M, et al. (2012): Annotation of functional variation in personal genomes using RegulomeDB. *Genome research*. 22:1790-1797.

4. Consortium EP (2012): An integrated encyclopedia of DNA elements in the human genome. *Nature*. 489:57-74.

5. Fullard JF, Giambartolomei C, Hauberg ME, Xu K, Voloudakis G, Shao Z, et al. (2017): Open chromatin profiling of human postmortem brain infers functional roles for non-coding schizophrenia loci. *Human molecular genetics*. 26:1942-1951.

6. de la Torre-Ubieta L, Stein JL, Won H, Opland CK, Liang D, Lu D, et al. (2018): The Dynamic Landscape of Open Chromatin during Human Cortical Neurogenesis. *Cell*. 172:289-304.e218.

7. Fullard JF, Hauberg ME, Bendl J, Egervari G, Cirnaru MD, Reach SM, et al. (2018): An atlas of chromatin accessibility in the adult human brain. *Genome research*. 28:1243-1252.

8. Bryois J, Garrett ME, Song L, Safi A, Giusti-Rodriguez P, Johnson GD, et al. (2018): Evaluation of chromatin accessibility in prefrontal cortex of individuals with schizophrenia. *Nature communications*. 9:3121.

9. Wang D, Liu S, Warrell J, Won H, Shi X, Navarro FCP, et al. (2018): Comprehensive functional genomic resource and integrative model for the human brain. *Science (New York, NY)*. 362.

10. Frankish A, Diekhans M, Ferreira AM, Johnson R, Jungreis I, Loveland J, et al. (2019): GENCODE reference annotation for the human and mouse genomes. *Nucleic acids research*. 47:D766-d773.

11. Abyzov A, Urban AE, Snyder M, Gerstein M (2011): CNVnator: an approach to discover, genotype, and characterize typical and atypical CNVs from family and population genome sequencing. *Genome research*. 21:974-984.

12. Zhu M, Need AC, Han Y, Ge D, Maia JM, Zhu Q, et al. (2012): Using ERDS to infer copy-number variants in high-coverage genomes. *American journal of human genetics*. 91:408-421.

13. Layer RM, Chiang C, Quinlan AR, Hall IM (2014): LUMPY: a probabilistic framework for structural variant discovery. *Genome biology*. 15:R84.

14. Chen X, Schulz-Trieglaff O, Shaw R, Barnes B, Schlesinger F, Kallberg M, et al. (2016): Manta: rapid detection of structural variants and indels for germline and cancer sequencing applications. *Bioinformatics*. 32:1220-1222.

15. Trost B, Walker S, Wang Z, Thiruvahindrapuram B, MacDonald JR, Sung WWL, et al. (2018): A Comprehensive Workflow for Read Depth-Based Identification of Copy-Number Variation from Whole-Genome Sequence Data. *American journal of human genetics*. 102:142-155.

16. Marshall CR, Howrigan DP, Merico D, Thiruvahindrapuram B, Wu W, Greer DS, et al. (2017): Contribution of copy number variants to schizophrenia from a genome-wide study of 41,321 subjects. *Nature genetics*. 49:27-35.

17. Rees E, Walters JT, Georgieva L, Isles AR, Chambert KD, Richards AL, et al. (2014): Analysis of copy number variations at 15 schizophrenia-associated loci. *The British journal of psychiatry : the journal of mental science*. 204:108-114.

18. Loh PR, Genovese G, Handsaker RE, Finucane HK, Reshef YA, Palamara PF, et al. (2018): Insights into clonal haematopoiesis from 8,342 mosaic chromosomal alterations. *Nature*. 559:350-355.

19. Dolzhenko E, van Vugt JJFA, Shaw RJ, Bekritsky MA, van Blitterswijk M, Narzisi G, et al. (2017): Detection of long repeat expansions from PCR-free whole-genome sequence data. *Genome Res*. 27:1895-1903.

20. Orr HT, Zoghbi HY (2007): Trinucleotide repeat disorders. *Annual review of neuroscience*. 30:575-621.

21. DeJesus-Hernandez M, Mackenzie IR, Boeve BF, Boxer AL, Baker M, Rutherford NJ, et al. (2011): Expanded GGGGCC hexanucleotide repeat in noncoding region of C9ORF72 causes chromosome 9p-linked FTD and ALS. *Neuron*. 72:245-256.

22. Chang SH, Gao L, Li Z, Zhang WN, Du Y, Wang J (2013): BDgene: a genetic database for bipolar disorder and its overlap with schizophrenia and major depressive disorder. *Biological psychiatry*. 74:727-733.

23. Wu Y, Yao YG, Luo XJ (2017): SZDB: A Database for Schizophrenia Genetic Research. *Schizophrenia bulletin*. 43:459-471.

24. Singh T, Poterba T, Curtis D, Akil H, Al Eissa M, Barchas JD, et al. (2020): Exome sequencing identifies rare coding variants in 10 genes which confer substantial risk for schizophrenia. *medRxiv*.2020.2009.2018.20192815.

25. Wu Y, Li X, Liu J, Luo XJ, Yao YG (2020): SZDB2.0: an updated comprehensive resource for schizophrenia research. *Human genetics*.

26. Orr HT, Zoghbi HY (2007): Trinucleotide repeat disorders. *Annu Rev Neurosci*. 30:575-621.

27. Pedersen BS, Quinlan AR (2017): Who's Who? Detecting and Resolving Sample Anomalies in Human DNA Sequencing Studies with Peddy. *American journal of human genetics*. 100:406-413.
